# Full-Genome Sequence of a Rare Human G3P[9] Rotavirus Strain

**DOI:** 10.1128/genomeA.00143-14

**Published:** 2014-03-27

**Authors:** Slavica Mijatovic-Rustempasic, Sunando Roy, Michele Sturgeon, Kunchala Rungsrisuriyachai, Mathew D. Esona, Dona DeGroat, Xuan Qin, Margaret M. Cortese, Michael D. Bowen

**Affiliations:** a Division of Viral Diseases, National Center for Immunization and Respiratory Diseases, Centers for Disease Control and Prevention, Atlanta, Georgia, USA; b Department of Laboratories & Pathology, Seattle Children’s Hospital, Seattle, Washington, USA

## Abstract

This is a report of the complete genomic sequence of a rare rotavirus group A G3-P[9]-I2-R2-C2-M2-A3-N2-T1-E2-H3 strain designated RVA/Human-wt/USA/12US1134/2012/G3P[9].

## GENOME ANNOUNCEMENT

Group A rotaviruses (RVAs) possess a segmented double-stranded RNA (dsRNA) genome composed of 11 segments encoding six structural proteins (VPs) and five or six nonstructural proteins (NSPs) (1). The classification nomenclature for the VP7, VP4, VP6, VP1-3, and NSP1-5/6 genes uses the notation Gx-P[x]-Ix-Rx-Cx-Mx-Ax-Nx-Tx-Ex-Hx, with x indicating the numbers of the corresponding genotypes (2). The majority of human RVA strains possess either the Wa-like genogroup 1 constellation (I1-R1-C1-M1-A1-N1-T1-E1-H1) or the DS-1-like genogroup 2 constellation (I2-R2-C2-M2-A2-N2-T2-E2-H2), which is believed to have origins with the porcine or the bovine RVAs, respectively (3). A smaller group of RVA strains have the AU-1-like genogroup 3 constellation (I3-R3-C3-M3-A3-N3-T3-E3-H3), which originated from feline RVAs (3, 4). Genogroup 3 RVAs cause rare human disease with limited transmission (5).

Here, we report the full-genome sequence of rotavirus strain RVA/Human-wt/USA/12US1134/2012/G3P[9] (12US1134), detected in a stool sample collected through the National Rotavirus Strain Surveillance System (7) from a 6-year-old who was treated at the Seattle’s Children Hospital, Seattle, WA, in 2012. Rotavirus dsRNA was extracted from the sample using TRIzol (Invitrogen), followed by DNase I treatment, and then was separated from single-stranded RNA by LiCl precipitation. The sequencing templates were prepared by using sequence-independent whole-genome reverse transcription-PCR (RT-PCR) amplification (8), with slight modifications. PCR amplicons were sequenced by the Illumina MiSeq 150 paired-end method by the Genomics Lab, HudsonAlpha Institute for Biotechnology, AL. Illumina sequence reads were analyzed using CLC Genomics Workbench 6.0. A combination of *de novo* assembly and subsequent mapping to a G3P[9] reference strain was used to obtain the full-length genome of strain 12US1134. The sizes of full-length segments 1 to 11 were 3,302, 2,687, 2,591, 2,359, 1,578, 1,356, 1,075, 1,059, 1,062, 751, and 667 bp, and the open reading frames (ORFs) for these segments were 3,267, 2,643, 2,508, 2,328, 1,476, 1,194, 942, 954, 981, 528, and 597 bp, respectively. The genotype assignment for each gene was accomplished using the RotaC online classification tool (http://rotac.regatools.be/) and BLAST (http://blast.ncbi.nlm.nih.gov/Blast.cgi).

The full-genotype constellation for strain 12US1134 is G3-P[9]-I2-R2-C2-M2-A3-N2-T1-E2-H3. This strain is nearly identical (98.9 to 99.8% nucleotide identity) to G3P[9] strain 0537, described previously (9), but only a partial sequence for strain 0537 was reported. This is the first complete RVA sequence with the genotype G3-P[9]-I2-R2-C2-M2-A3-N2-T1-E2-H3.

Several full-genome sequences of G3P[9] RVA strains of feline or human origin have been reported (6, 10). G3P[9] strains of human origin (PAI58/96, PAH136/96, and 0537) have a relatively stable genetic constellation, G3-P[9]-I2-R2-C2-M2-A3-(N1/N2)-(T1/T6)-E2-H3, and carry a DS-1 backbone, which differentiates them from most G3P[9] strains of feline origin.

Full-genome studies help expand our knowledge of the genetic diversity and origin of uncommon rotavirus genotypes and highlight the need for continuous monitoring of RVA strains for timely recognition of novel or rare genotypes, including in postvaccine introduction settings.

### Nucleotide sequence accession numbers.

The full-genomic sequence of the G3P[9] rotavirus strain 12US1134 has been deposited in GenBank under accession no. KF500514 to KF500524.

## References

[1] Estes MK Kapikian AZ 2007 Rotaviruses, p 1917–1974 *In* Knipe DM Howley PM Griffin DE Lamb RA Martin MA Roizman B Straus SE (ed), Fields virology, 5th ed, vol 2. Lippincott Williams & Wilkins, Philadelphia, PA.

[2] Matthijnssens J Ciarlet M McDonald SM Attoui H Bányai K Brister JR Buesa J Esona MD Estes MK Gentsch JR Iturriza-Gómara M Johne R Kirkwood CD Martella V Mertens PP Nakagomi O Parreño V Rahman M Ruggeri FM Saif LJ Santos N Steyer A Taniguchi K Patton JT Desselberger U Van Ranst M 2011 Uniformity of rotavirus strain nomenclature proposed by the Rotavirus Classification Working Group (RCWG). Arch. Virol. 156:1397–1413. 10.1007/s00705-011-1006-z 21597953 PMC3398998

[3] Matthijnssens J Ciarlet M Heiman E Arijs I Delbeke T McDonald SM Palombo EA Iturriza-Gómara M Maes P Patton JT Rahman M Van Ranst M 2008 Full genome-based classification of rotaviruses reveals a common origin between human Wa-Like and porcine rotavirus strains and human DS-1-like and bovine rotavirus strains. J. Virol. 82:3204–3219. 10.1128/JVI.02257-07 18216098 PMC2268446

[4] Matthijnssens J Taraporewala ZF Yang H Rao S Yuan L Cao D Hoshino Y Mertens PP Carner GR McNeal M Sestak K Van Ranst M Patton JT 2010 Simian rotaviruses possess divergent gene constellations that originated from interspecies transmission and reassortment. J. Virol. 84:2013–2026. 10.1128/JVI.02081-09 19939934 PMC2812371

[5] Matthijnssens J Van Ranst M 2012 Genotype constellation and evolution of group A rotaviruses infecting humans. Curr. Opin. Virol. 2:426–433. 10.1016/j.coviro.2012.04.007 22683209

[6] De Grazia S Giammanco GM Potgieter CA Matthijnssens J Banyai K Platia MA Colomba C Martella V 2010 Unusual assortment of segments in 2 rare human rotavirus genomes. Emerg. Infect. Dis. 16:859–862. 10.3201/eid1605.091826 20409385 PMC2954010

[7] Hull JJ Teel EN Kerin TK Freeman MM Esona MD Gentsch JR Cortese MM Parashar UD Glass RI Bowen MD National Rotavirus Strain Surveillance System National Rotavirus Strain Surveillance S 2011 United States rotavirus strain surveillance from 2005 to 2008: genotype prevalence before and after vaccine introduction. Pediatr. Infect. Dis. J. 30:S42–S47. 10.1097/INF.0b013e3181fefd78 21183839

[8] Potgieter AC Page NA Liebenberg J Wright IM Landt O van Dijk AA 2009 Improved strategies for sequence-independent amplification and sequencing of viral double-stranded RNA genomes. J. Gen. Virol. 90:1423–1432. 10.1099/vir.0.009381-0 19264638

[9] Grant L Esona M Gentsch J Watt J Reid R Weatherholtz R Santosham M Parashar U O’Brien K 2011 Detection of G3P[3] and G3P[9] rotavirus strains in American Indian children with evidence of gene reassortment between human and animal rotaviruses. J. Med. Virol. 83:1288–1299. 10.1002/jmv.22076 21567432

[10] Tsugawa T Hoshino Y 2008 Whole genome sequence and phylogenetic analyses reveal human rotavirus G3P[3] strains Ro1845 and HCR3A are examples of direct virion transmission of canine/feline rotaviruses to humans. Virology 380:344–353. 10.1016/j.virol.2008.07.041 18789808 PMC2575048

